# Evaluation of Physico-Mechanical Properties and Thermal Conduction to Energy-Saving Effects of Wood Compression Layered Structural Materials

**DOI:** 10.3390/polym15153208

**Published:** 2023-07-28

**Authors:** Yu-Hui Chen, Shang-Tse Ho, Han-Chien Lin

**Affiliations:** Department of Wood Based Materials and Design, National Chiayi University, Chiayi 600, Taiwan; naomiqq75@gmail.com (Y.-H.C.); stho@mail.ncyu.edu.tw (S.-T.H.)

**Keywords:** wood compression layered structural materials (WCLS), densification, dimensional stability, thermal conductivity, energy-saving effect

## Abstract

In this study, specimens were prepared from Japanese cedar (*Cryptomeria japonica*) with different thicknesses to determine the best hot-pressing conditions for wood compression layered structural materials (WCLS) through densification at various temperatures and compressing time conditions. However, residual stress-releasing after densification recovery can cause dimensional instability. To address this issue, the drying set method was combined with the compression-set recovery test to determine the best setting time. As a result, the bending strength and modulus of rupture (MOR) of WCLS increased by 9.98 ± 9.71 to 20.87 ± 13.09% and the modulus of elasticity (MOE) increased by 9.87 ± 11.92 to 22.40 ± 17.97%. The volumetric swelling coefficient (S), water absorption percent (WAP), and equivalent moisture content (EMC) decreased as the drying time increased. The anti-swelling efficiency (ASE) and moisture excluding efficiency (MEE) were found to be the highest at a drying time of 12 h, with values ranging from 13.20 ± 15.11 to 36.46 ± 6.83% and 15.18 ± 1.11 to 19.58 ± 8.31%, respectively. The drying method was found to be effective in increasing dimensional stability. The glass transition temperature (*Tg*) moved to a lower temperature as the compression-set increased, which was due to plasticization of wood caused by high temperature and pressure. The cell walls of WCLS presented viscous buckling, which provided effective dimensional stability. The thermal conductivity of Japanese cedar and each compression-set WCLS were 0.1863 ± 0.0071, 0.1520 ± 0.0147, 0.1817 ± 0.0106, and 0.1423 ± 0.0137 W/mK, respectively. The thermal conductivity of each WCLS increased with an increase in compression-set, but decreased by 10.67 to 22.52% compared to Japanese cedar. The total electricity energy consumption of each WCLS after 24 h of testing decreased with a trend of 30.50 ± 0.84, 29.83 ± 0.42, 29.57 ± 0.51, and 29.4 ± 0.36 kWH.

## 1. Introduction

Taiwan relies heavily on imported timber and its annual self-sufficiency rate is less than 1% of its import rate, despite being rich in forests, according to the fourth survey of the Forestry Bureau [1]. Enhancing the utilization of Japanese cedar (*Cryptomeria japonica*), which comprises approximately 40% of planted coniferous forests, is crucial for promoting domestic timber usage in Taiwan. However, the domestically available Japanese cedar is primarily composed of small-diameter, lightweight, and with numerous knots. The low density of Japanese cedar leads to low surface hardness and wear resistance, limiting its potential applications. To address this issue, one approach to enhance the utilization of Japanese cedar is by improving its properties using modification methods. Chemical modification methods, such as wood acetylation, furfuralization, and impregnation with thermosetting resins, can improve dimensional stability, durability, and reduce wood biodegradation [2,3,4,5]. However, chemical modification methods may also influence both the environment and human health. Physical modification methods, such as high temperature and high pressure, can be used as an alternative with lower environment impact. Those methods change the composition of the wood and improve its utilization without the use of chemical agents [6].

Among the methods of high temperature and high pressure, the technology of compressed wood has existed for a long time, with patents issued in the USA and Europe [7]. Morsing and Hoffmeyer [8] reported that the technology of wood densification can increase its application value. The densification process is divided into following steps. Firstly, the cell wall is softened or plasticized. Secondly, the wood specimen is compressed in the direction of the vertical grain while in a softened state. Thirdly, the compressed wood specimen is cooled while in a deformed state, allowing it to set into its new shape. However, densified wood tends to recover after absorbing moisture. This is due to the release of stress stored in the crystallized area of the microfiber, caused by the mechanical high pressure. Approximately 50% of the maximum total residual stress of compressed wood is released after it is immersed in hot water [9,10]. Inoue et al. [11,12,13] reported that reducing recovery by changing the hygroscopicity of the wood cells, forming covalent crosslinks in the deformed state, can reduce stress release. Esteves and Pereira [14], Dwianto et al. [15], and Zhang [16] indicated that heat treatment can effectively reduce cell recovery due to the bond breaking of wood hemicellulose and the slight cracking of lignin.

The strength of compressing wood is also improved compared to the uncompressed wood. This may contribute to the increase in wood density. Both the flexural modulus of elasticity and the flexural strength increased [17,18,19]. However, Blomberg et al. [20], Ulker et al. [21], and Lu [22] have pointed out that during the densification process, as the temperature increases, the degradation of wood components also increases, which may result in lower mechanical strength. Müller et al. [23], Zhang [16], and Kutnar et al. [24] have also mentioned that the deformation of compressed wood is a transitional period from elastic to plastic deformation, and the wood cells are in a rubbery state after deformation. This step plays important role in preventing wood breaking. Through microscopic analysis, the volume of the intracellular void area is uniformly reduced and then improved the mechanical properties of compressed wood [25]. In addition, wood densification further affected wood properties include moisture content, specific gravity, and annual ring angle, etc. These differences are related to the microstructure of the cell wall and the degree of plasticity during cell compression [16,23].

To prevent wood from recovering after compression, cross-linking can be formed between the wood components in a deformed state by changing the hygroscopicity of the cells [17]. During heat treatment, the thermal degradation of hemicellulose and cellulose reduces the number of hydrophilic OH groups, which increases the crystallinity of cellulose and decreases water absorption of wood. This significantly impacts the moisture resistance of wood by reducing the equilibrium moisture content and effectively solving the recovery of compressed wood due to moisture absorption [6,26]. In the heat treatment method, the drying-set treatment effectively reduces recovery by using stable temperature, continuous pressure, and long-term drying. This gradually reduces the moisture content of the wood and the storage stress in the cells [27]. Additionally, after heat treatment, the anti-corrosion and anti-biological invasion properties of treated wood are enhanced, and the wood surface color becomes darker, which reduces the color difference between sapwood and heartwood [28,29].

The global greenhouse effect is becoming increasingly serious and the CO_2_ content is rising year by year. With the growing awareness of environmental protection, energy conservation has become an important topic for the related field. Wood is an effective insulator due to its low thermal conductivity, and its porous nature with moisture absorption and desorption properties enables it to regulate temperature and humidity simultaneously. Wang [30] and Liao [31] have mentioned that wood, when used as building exterior and interior materials, can help regulate the indoor temperature. Ventilation, when coupled with wood, can effectively reduce indoor humidity, thereby maintaining indoor temperature and humidity [32,33]. Liao [31] and Wang and Li [34] reported that when wood is used in combination with cement walls as building decoration interior, it can save about 11% of electricity consumption with using air conditioner. The thermal conductivity of air is lower than that of water, which means that porosity indirectly affects the thermal conductivity of wood. Thus, wood with low density or thicker thickness has higher porosity, and if the content of air is increased, the thermal conductivity will be decreased. However, when the moisture content of wood increases, it will lead to an upward trend [31,35]. Seo et al. [36] mentioned that the thermal conductivity of the floor is related to the material density, and the thermal conductivity of wood materials increases with the increase of density.

This study aimed to densify Japanese cedar in order to improve its light and soft texture and transform it into high-strength wood. To address the issue of stress stored in the cells during the compression process leading to recovery problems, a drying-set method was employed to develop wood compression layered structural materials (WCLS). WCLS possess higher strength and dimensional stability, and reduce the recovery caused by re-absorbing moisture, thereby producing a more durable and stable product. The properties of WCLS obtained after physical processing was investigated. The present study provides important information for further related researches and the relevant wood processing industry. The development of WCLS not only enhances the utilization of domestic Japanese cedar in Taiwan but also expands the potential applications and value of Japanese cedar.

## 2. Materials and Methods

### 2.1. Materials

36 years old Japanese cedar (*Cryptomeria japonica*) was provided by Chiayi Forest District Office. The test materials were prepared as longitudinal length of 325 mm, a tangential width of 65 mm, and a radial thickness of 22, 24, and 27 mm. The wood was selected to ensure that there were no natural defects such as knots or cracks. As shown in Figure S1, test specimens were further selected based on an annual ring inclination angle of 61–90°, as determined by the cross-section of the annual rings [16]. All of the experimental materials are of analytical grade.

### 2.2. Basic Properties Measurement of Japanese Cedar

#### 2.2.1. Moisture Content

CNS 452 [37] was used to determine the moisture content of the wood. The test material was placed in an oven at a temperature of 103 ± 2 °C and dried to a constant weight. After drying, the test material was taken out of the oven and weighed. The moisture content was then calculated using Equation (1).

Moisture content (%) = (m^1^ − m^2^/m^2^) × 100 (1)

m^1^ and m^2^ were weight of a wood specimen before and after oven-dried, respectively.

#### 2.2.2. Density

The test method for wood density followed the standard CNS 451 [38]. Test specimens were randomly selected and the absolute dry weight and volume were measured. Wood density was calculated using the Equation (2). The density increase rate was then calculated from the absolute dry density of the compressed wood before and after treatment under various conditions.

ρ (kg/m^3^) = m/v (2)

ρ, m and v represent the density, mass, and volume of the specimen, respectively.

### 2.3. Development of WCLS

#### 2.3.1. Pretreatment of Test Material before Compression

Japanese cedar logs were cut into segments at the HF lumber factory (Chiayi, Taiwan). The preparation method of test specimens were shown in Figure S2. The resulting board size was approximately 6 feet in length, 0.22 feet in width, and 1 inch in thickness (approximately 1820 × 67 × 30 mm).

The boards prepared by the HF lumber factory were stacked using a flat stacking method and placed in the solar dehumidification dry kiln at the environmental functional materials research lab of the department of wood-based materials and design, National Chiayi University. After drying, the specimens were cut and placed in a constant temperature and humidity room with a temperature of 20 °C and a relative humidity of 65% for more than 3 weeks. Once the moisture content reached an equilibrium state of 10–12%, the test specimen was prepared as shown in Figure S3 for further experiment.

After conditioning, the specimens were planed to a thickness of 22, 24, and 27 mm which were measured using a digital caliper. Additionally, to prevent bursting during the hot-pressing process and maintain a lower moisture content (4–6%), Japanese cedar with an equilibrium moisture content of 10–12% after humidity adjustment was used. The specimens were then placed on a hot press machine. The upper and lower plates of the hot press were brought close to the specimens to preheat them without touching. After preheating, the moisture content of the specimens was measured under different preheating temperature conditions using the moisture content meter HM 500. At a preheating temperature of 160 °C, it took 27 min of preheating to achieve a low moisture content of 4–6%. At a preheating temperature of 180 °C, the preheating time needed to be at least 23 min. When the temperature reached 200 °C, the time required was approximately 17 min. The specimens were subjected to one-stage hot pressing after preheating, with a fixed pressing speed of 12 mm/s. As shown in Table 1, the following conditions were adopted for hot pressing.

#### 2.3.2. Selecting Conditions for Densification

The thickness of the wood specimens was measured at 2 points on the top, middle, and bottom before and after hot pressing under each condition. After the final measurement, the specimens were placed in a constant temperature and humidity room with an ambient temperature of 20 °C and a relative humidity of 65%. The thickness change at the marked positions was recorded every 12 h, and the set-recovery of the compressed Japanese cedar specimens was observed after being left for 72 h. Equation (3) was used to calculate the set-recovery.

Set-recovery (%) = [(L_1_ − L_c_)/(L_0_ − L_c_)] × 100 (3)

L_0_ and L_c_ represent the thickness of the wood specimen before and after compression, respectively, and L_1_ represents the thickness after placing the specimen in the constant temperature and humidity chamber for 72 h.

#### 2.3.3. Development of Drying-Set Treatment

The drying-set treatment method used in this study involved the long-term pressurization and drying method proposed by Sandberg and Johansson [27] to stabilize the dimensional stability of the densified material. The densified Japanese cedar specimens with different compression levels were prepared. The densification of the Japanese cedar specimens was maintained at a target thickness of 18 mm by sequentially tightening the 12 nuts with a torque wrench to 450 kgf·cm until the stainless plate was close to the stop bar. The specimen was then fixed in the drying-set mold as shown in Figure S4. The entire set of drying-set mold modules was placed in an oven at a temperature of 100 °C for 6, 12, and 24 h for drying-set, and obtained the resulting WCLS.

To investigate the compression-set recovery (Crsp) of WCLS after water immersion, the following process was performed: three cycles of water immersion were carried out within one week using 20 mm specimens on the radial (L) and tangential (W) sections. The cycle process consisted of placing the specimens in an oven at 103 ± 2 °C and drying them overnight to obtain the initial completely dry size of the specimens. Then, the specimens were immersed in water at 20 °C for 3.5 h and subsequently dried in an oven until completely dry. After each cycle, the weight and thickness of the specimens were measured, and the Crsp of WCLS was calculated using Equation (4) [12,17].

Crsp (%) = [(Lcycle − Lafc)/(L0cr − Lafc)] × 100(4)

L0cr, Lafc and Lcycle were the dry thickness of Japanese cedar specimens before compression, the thickness of WCLS, and the thickness after each cycle of immersion, respectively.

### 2.4. Properties Determinate of WCLS

After densification combined with drying-set, the treatment of WCLS was shown in Table 2.

#### 2.4.1. Basic Properties Measurement of WCLS

WCLS was developed by densification with different compression ratios combined with drying-set treatment. After measuring the thickness of the middle and both sides, and the compression-set (Cr) was calculated by Equation (5) [17].

Cr (%) = (L_0_ − Lafc)/L_0_ × 100 (5)

L_0_ and Lafc were initial thickness of specimens, and thickness after densification and drying-set, respectively.

To determine the weight loss of specimens after hot pressing and drying-set treatment, the specimens weight was measured. The testing method involved weighing the absolute dry weight (W_0_) of the Japanese cedar specimens before compression. The specimens were then densified under different conditions combined with drying-set treatment to develop WCLS, and dried in the oven. The mass (W_1_) of the resulting WCLS was weighed after the temperature dropped to room temperature, with 6 repetitions performed. Weight loss (WL) was calculated using Equation (6).

WL (%) = (W_0_ − W_1_)/W0 × 100 (6)

W_0_ and W_1_ were initial weight of specimens, and weight after densification and drying-set respectively.

To obtain specimens for density profile, WCLS specimens with different compression-set were cut in half along the longitudinal direction from the original size of 325 × 65 × 18 mm to create specimens of size 325 × 30 × 18 mm. The surface of each specimen was then sanded using a broadband sander, starting with 1 mm grit and decreasing in increments to 0.5 mm and then 0.1 mm until the specimen was less than half its original thickness. After each sanding, the weight and thickness of the specimen were measured at a fixed position, and the density was calculated using Equation (7). The calculated data for each layer were used to create a density profile. This testing method is based on the procedure described by Shiu et al. [39].

ΔD (g/cm^3^) = [(W_1_ − W_2_)/(L × W × ΔH)] × 100 (7)

W_1_, W_2_ and ΔH were weight before and after sanding of WCLS and thickness before and after sanding of WCLS, respectively.

#### 2.4.2. Surface Properties Measurement of WCLS

The testing process involved sticking each specimen (10 × 2 × 3 mm) on the aluminum platform with a diameter of 14 mm and a thickness of 8 mm, and analyzed using scanning electron microscope (TM 3000) to observe their cellular structure.

#### 2.4.3. Measurement of Physico-Mechanical Properties of WCLS

The experimental procedure for measuring the swelling behavior of the specimens is described as follows. First, the volume and weight of the compressed specimen and WCLS (20 × 20 × 18 mm) were measured under dry conditions. The specimens were then immersed in distilled water, evacuated for 30 min, returned to normal pressure, and maintained for 1 h. This process was repeated, with a 30-min evacuation and 1-h maintenance period after each immersion in water, until the end of day 7. At this point, the volume and weight of the specimens were measured under wet conditions. This procedure was repeated six times for each condition. This experimental method was adapted from Kuo and Lu [6].

After each specimen was soaked in water, the volumetric swelling coefficient (S) was calculated using Equation (8), the antiswelling efficiency (ASE) was calculated using Equation (9), and the water absorption percent (WAP) was calculated using Equation (10).

S (%) = ((V_w_ − V_0_)/V_0_) × 100 (8)

V_w_ and V_0_ were volume in saturated state and oven-dried state of WCLS, respectively.

ASE (%) = ((S_c_ − S_t_)/S_c_) × 100 (9)

S_c_ and S_t_ were volumetric swelling coefficient of compressed specimens and WCLS, respectively.

WAP (%) = ((W_w_ − W_0_)/W_0_) × 100 (10)

W_w_ and W_0_ were mass in saturated state and oven-dried weight of WCLS, respectively.

Each WCLS and compressed specimen were cut to the size required for the hygroscopicity test, with dimensions of 20 × 20 mm for length and width, and the same thickness as the original test material. They were dried in an oven at 103 ± 2 °C until their weight reached a constant value, and then moved to a constant temperature and humidity chamber with a temperature of 40 °C and a relative humidity of 65% to adjust the humidity. After conditioning for 72 h, the weight of the specimens was measured, and Equations (11) and (12) were used to calculate the equivalent moisture content (EMC) and moisture excluding efficiency (MEE) of the WCLS at a relative humidity of 65%. The specimens were then moved to a constant temperature and humidity chamber with a temperature of 40 °C and a relative humidity of 95% to condition. After another 72 h, the weight of the specimens was measured, and the EMC (%) and MEE of the specimens in an environment with a relative humidity of 95% were calculated. The tests were repeated six times for each different thickness condition [6].

EMC (%) = ((W_E_ − W_0_)/W_0_) × 100 (11)

W_E_ and W_0_ were mass of specimen when it is equilibrated at the relative humidity and oven-dried weight of specimen, respectively.

MEE (%) = ((Ec − Et)/Ec) × 100(12)

Ec and Et were equivalent moisture content of compressed specimens and WCLS, respectively.

The test method of bending strength referred to CNS 3904 [40] and CNS 9907 [41]. Each test material was placed in a constant temperature and humidity room with 20 °C and RH 65% for more than 3 weeks. The size of WCLS and control group was 32.0 × 6.5 × 1.8 cm. The span of the test condition was 32.5 cm, the central concentrated loading method was adopted, the loading surface was tangential section, and the average loading speed was 3 mm/min. The Equation (13) was used to calculate the resistance modulus of rupture (MOR) and the Equation (14) to calculate modulus of elasticity (MOE). The increase rate was calculated by comparing the individual test results with the average result of the control group.

MOR (N/mm^2^) = 3PL/2bt^2^ (13)

MOE (N/mm^2^) = ΔPL^3^/4bt^3^Δd (14)

P, L, Δd, ΔP, b and t were maximum load (N), span (mm), the increase of deflection of linear part (mm), the increase of load of linear part (N), width of specimen (mm) and thickness of specimen (mm), respectively.

The CNS 460 [42] was used for hardness testing, and the EN1534 [43] wood was used for surface hardness testing, steel balls with a diameter of 10 ± 0.1 mm were selected and passed through a universal testing machine for measurement using the Brinell hardness method to measure resistance to dents. During the test, the average loading speed was 0.5 mm/min, and the indentation depth was 1/π (about 0.32 mm). The size of the specimen with different compression-set rates was 32.0 × 6.5 cm in length and width, and the measurement position was on the tangential section of the surface that had undergone a drying-set treatment after hot pressing. 10 positions were taken from each specimen for measurement, and 6 repeated test specimens were carried out under different conditions. The hardness was calculated using Equation (15), and the increase rate was determined by comparing the individual test result with the average result of the control group.

H(N) = ρ/10(15)

H and ρ were surface hardness and the maximum load when the steel ball was pressed into 1/π, respectively.

#### 2.4.4. Measurement of Thermal Properties of WCLS

The thermogravimetric analyzer (TGA) of PerkinElmer (Pyris 1 TGA, USA) was used to measure the t thermal properties of test specimens. The specimen was prepared by crushing the Japanese cedar into powder and then using a sieve to obtain granules with a diameter less than 60 mesh. Five milligrams of the specimen were taken. The test process was carried out in a nitrogen environment with a heating rate of 10 °C/min as the test condition, and the measured temperature range was 30–600 °C [44].

The glass transition temperature (*Tg*) test was analyzed by a PerkinElmer DMA 7 e dynamic mechanical analyzer (DMA). The test process adopted three-point bending mode, and the test piece was placed in the mold with a size of 14 × 3 × 3 mm. The test conditions were a heating rate of 3 °C/min and a frequency of 1.2 Hz, and the measured temperature range was 150–250 °C. Through the storage modulus (E′) and loss modulus (E″) data measured during the test, it was analyzed that when temperature increased, the internal state of the specimen changed from elastic to viscous. The obtained data of E′ were divided by E″ to obtain the data of loss tangent (Tan δ), and the peak value was observed to understand *Tg* between WCLS and control group.

#### 2.4.5. Evaluation of Thermal Conductivity and Electricity Energy Consumption

The thermal conductivity of WCLS with different development conditions was measured in atmospheric environment in a closed space after being taken out of a constant temperature and humidity chamber with 25 °C and RH 65%. The temperature and humidity in the atmospheric environment were recorded by a temperature and humidity recorder (HOBO U23 Pro-v2). To record the change of heat flow (kcal/m^2^ h) through a portable heat flow meter (HFM-201), the thermal conductivity of the specimen was calculated using Equation (16). The purpose of this measurement was to investigate the thermal conductivity of WCLS in the atmosphere environment.

Thermal conductivity (λ) = q × S/A × t × ΔT = Q × S/ΔT(16)

q, S, A, t, ΔT and Q were energy of specimen, thickness of specimen (m), surface area (m^2^), per unit time (s), θ_2_−θ_1_ (°C) and heat flow (kcal/m^2^ h), respectively.

The thermal conductivity of each specimen was measured in units of kcal/m^2^ h, which represents the heat flow density. However, previous studies by Asako et al. [45] and Seo et al. [36] reported thermal conductivity in units of W/mK. To convert between these units, we used Equation (17), where W (Watt) is the energy consumption rate per unit time and kcal/h is converted to 1.16299 W. The temperature used in the conversion was 273.15 K at 0 °C, and since Celsius temperature (°C) is equal to Kelvin (K), the temperature difference can be expressed in Kelvin. The following test results are presented in both units for comparison.

Thermal conductivity (kcal/mh °C) × 1.16299 = λ(W/mK)(17)

W, m and K were the energy consumed by specimen per unit time, thickness of specimen (m) and absolute temperature (K), respectively.

To evaluate the thermal conductivity and electricity energy consumption of WCLS with different development conditions, we placed the specimens in a device that simulated indoor and outdoor environments. The thermal conductivity and electricity energy consumption of the specimens were observed in different simulated environments using a portable heat flow meter (HFM-201), a three-phase power analyzer (DW-6092), a memory four-window thermometer (TM-947SD), and a temperature and humidity recorder (HOBO U23 Pro-v2). Each specimen (325 × 60 × 18 mm) was placed in the test box of the simulated environment. A wall board was used to simulate the isolation of indoor and outdoor temperature and humidity. The test box, made of acrylic, had an opening area of 720 × 550 mm and an overall size of 720 × 715 × 550 mm. It was used as a connection between two constant temperature and humidity machines (Taiqi Technology Co., Ltd., Huizhou, China) to simulate the intermediate connection between indoor and outdoor environments. The hot side of the simulated outdoor temperature was simulated by a programmable constant temperature and humidity machine (MHG-20RT), and the set temperature was 50 °C with relative humidity of 50, 60, 70, 80 and 90%. The cold side of the simulated indoor comfortable environment was simulated by a constant temperature and humidity machine (HRM-120R), and the temperature and humidity were set at 25 °C and 65%, respectively. To observe the thermal conductivity and electricity energy consumption of the specimens, we used a portable heat flow meter (HFM-201), a three-phase power analyzer (DW-6092), a memory four-window thermometer (TM-947SD), and a temperature and humidity recorder (HOBO U23 Pro-v2) [46].

The electricity energy consumption was measured using the single-phase two-wire (1Φ 2W) mode of the three-phase power analyzer (DW-6092) manufactured by Lutron Lu-chang Electronics Enterprise Co., Ltd., Wan Chai, Hong Kong. The instantaneous current (A) and volts (V) of the cold side constant temperature and humidity chamber were recorded and converted into power (Kw). The electricity energy consumption (kwH) was recorded while 2 h of heat flow stabilized during the test process, and the energy-saving effect of the specimen under different development conditions was evaluated.

To understand the influence of temperature and humidity changed in four seasons in Taiwan on the thermal conductivity and power saving benefits of WCLS, the monthly average temperature and humidity in Taiwan from 2011 to 2021 was used as the test conditions, and through Taipei, Taichung, Chiayi, Kaohsiung, Hualien as a representative city in Taiwan which recorded the temperature and humidity of these five cities in the past ten years. The data was collected by the observation data query system CODIS [47] which the lowest temperature of 14–17 °C, the highest temperature of 30–39 °C, the lowest relative humidity of 59–65% and maximum humidity 83–96%.

### 2.5. Statistical Analysis

The results of the basic and surface properties of WCLS are expressed as mean values (standard deviations). The software SPSS 12 (Statistical Product and Service Solutions) was used for Duncan’s multivariate domain analysis. *p* < 0.05 were considered to be significant.

## 3. Results and Discussion

### 3.1. Basic Properties of Japanese Cedar

**Figure 1:**
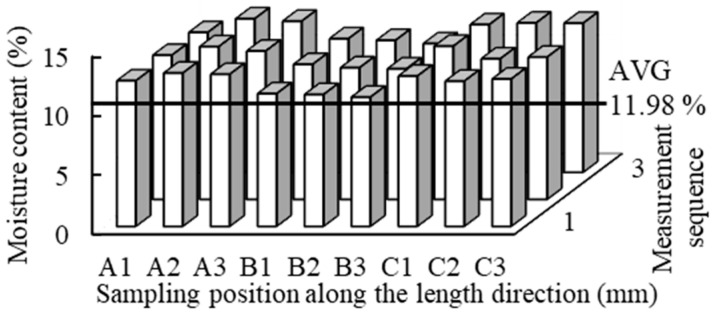
Distribution of moisture content of specimens.

The moisture content distribution of the specimens was measured (Figure 1). The average moisture content of the outer area A was 12.55%, while the average moisture content of the middle area B was 11.10%, and that of the outer area C was 12.41%. The average moisture content of the test material was 11.98%. Table 3 shows that the moisture content of Japanese cedar with different thicknesses ranged from about 9.77 to 10.78%, and there was no significant difference in the moisture content. The different thicknesses of Japanese cedar had densities ranging from about 370 to 420 kg/m^3^, and there was no significant difference. The air-dried moisture content of the wood in Taiwan was about 15%, and the equilibrium moisture content was about 10–12%. When the wood moisture content was at 12%, there was a 3.6% increase in longitudinal compressive strength for every 1% increase, which was more than that in the dry state [48].

### 3.2. Surface Densification

#### 3.2.1. Measurement of Hot-Pressing Temperature

**Figure 2:**
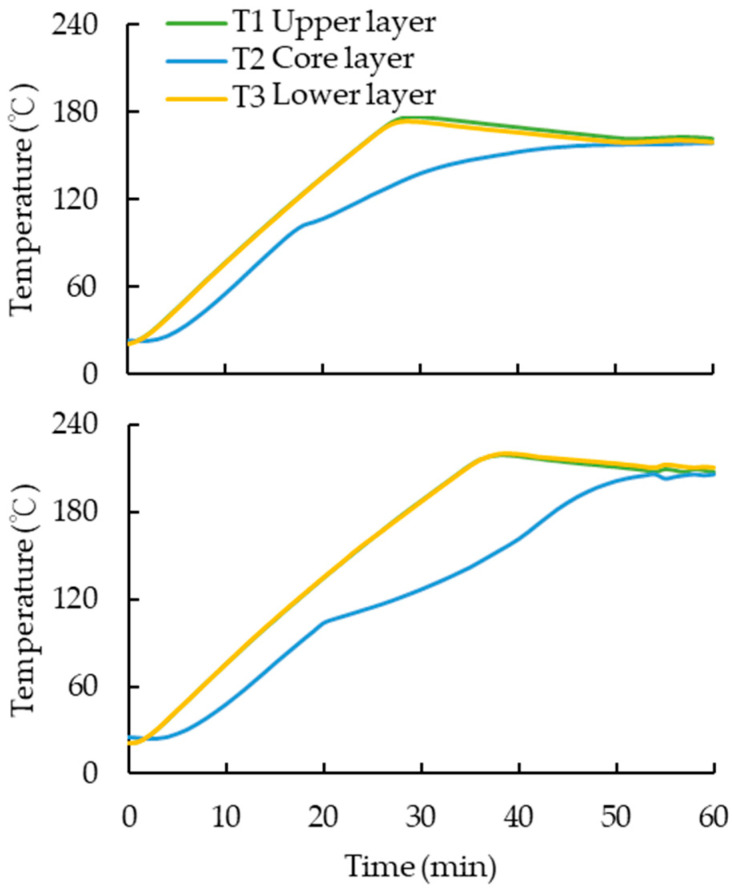
Temperature of specimen from surface layer to core layer. Up: hot-pressed at 160 °C for 60 min; Down: hot-pressed at 200 °C for 60 min.

In the process of hot pressing, the temperature is transmitted from the surface layer to the core layer to achieve the softening effect of the wood. As the temperature increases, the wood material thermally softens, and at the glass transition temperature (*Tg*), it changes from an elastic state to a plastic state, depending on the cellulose, hemicellulose, and lignin content and their respective softening conditions, which are affected by temperature and moisture content [49]. Hemicellulose changes first at 167–217 °C, followed by lignin at 134–235 °C, and cellulose at 231–253 °C [16,49]. The specimens with a thickness of 22 mm before compression were hot-pressed at 160 °C for 60 min, while specimens with a thickness of 27 mm before compression were hot-pressed at 200 °C for 60 min. The time required for the surface temperature of both specimens to pass into the core layer was determined. Figure 2 showed that it took more than 50 min for the temperature of the outer layer to gradually spread into the core layer during hot pressing. By prolonging the heating time, the specimens could reach near *Tg* and become plastic. These changes are due to the breaking of hemicellulose bonds when the wood is heated, which reduces the hydrophilic OH groups and causes slight degradayion of lignin as well [15]. In Figure 2, T1, T2, and T3 refer to layer of specimen that close to upper panel of hot press, the core layer of surface and specimen that close to lower panel of hot press.

#### 3.2.2. Compression-Set of Surface Densification

Different hot-pressing conditions significantly influenced the compression-set. Various conditions including hot-pressing temperatures (160, 180, and 200 °C), treatment time (30 and 60 min), and thicknesses of 22, 24, and 27 mm with different pressures were used for hot-pressing. The thickness compression-set decreased with the increase of hot-pressing time, and the higher hot-pressing temperature also had a similar effect. When wood is heated, wood components may be degraded due to the increase in temperature, resulting in a reduction of hydrophilic groups, increasing the hydrophobicity of wood and changing the hygroscopicity of cells [6,17,22], thereby effectively reducing the compression-set. Considering the results and energy-saving effect, the hot-pressing temperature of 180 °C was selected as the optimum hot-pressing temperature condition for the test.

Huang [50] reported that as the compressed thickness increased, the internal storage stress of the wood increased with the compression rate. When compressing specimens with different thicknesses, it was found that the rebound rate became more apparent with higher compression thickness. The thicker the compression thickness, the higher the compression rate that can be achieved, especially for the compression thickness of 27 mm (compression rate 33%), which was hot-pressed at 160 °C for 30 min, reaching a compression-set of 2.70%. This result shows that during the compression process, compressive stress is stored inside the wood, and after the wood is decompressed, the residual stress is released. The mechanically induced residual stress is enhanced with an increase in the compression rate [9].

**Figure 3:**
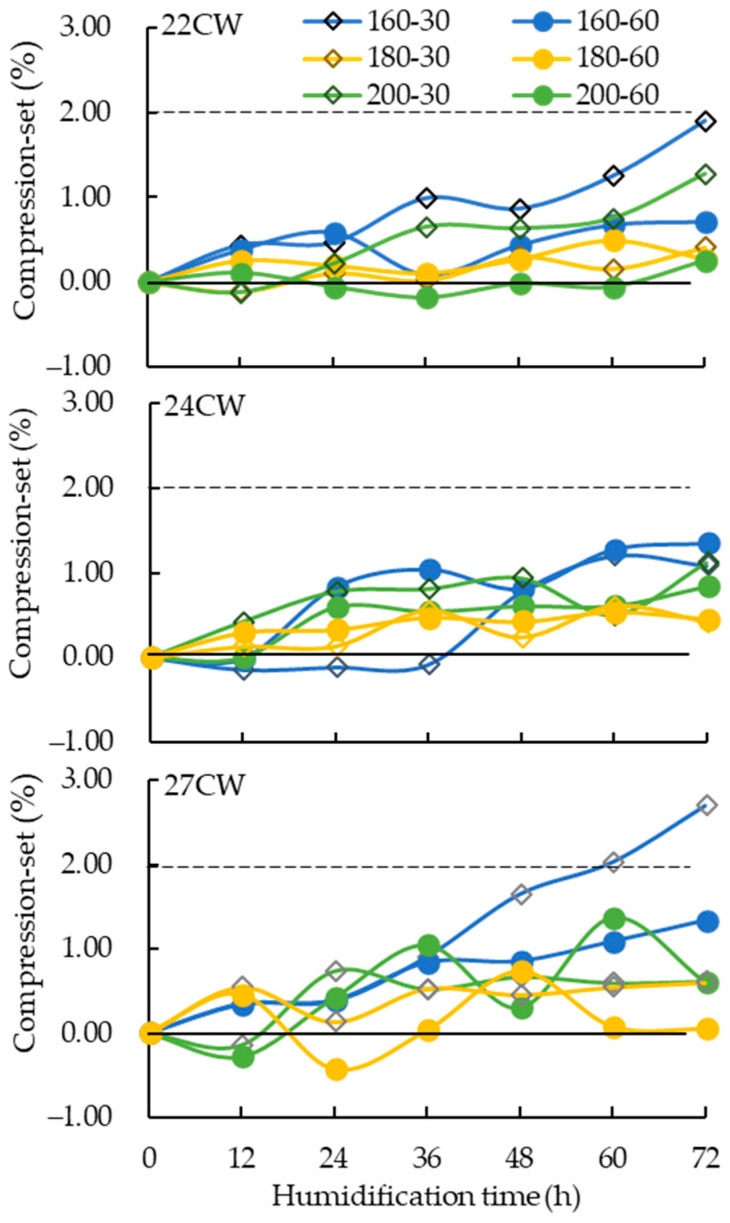
Compression−set of specimens with different manufacturing conditions.

Huang and Cho [50] indicated that increasing the temperature and time can effectively reduce the recovery rate of compressed specimens. As shown in Figure 3, under the same thickness and hot-pressing temperature, a longer compression time resulted in lower compression-set. This is because when the specimens were heated for a longer time, the temperature gradually spread from the surface to the core layer, softening both the inside and outside and reducing compression-set. After 30 min of hot-pressing, the compression-set for specimens with thicknesses of 22 mm, 24 mm, and 27 mm were 1.08%, 1.89%, and 2.39%, respectively. However, after 60 min of hot-pressing, these values decreased to 0.71%, 1.34%, and 1.35%, respectively, resulting in an average reduction of 35%. Additionally, the treatment time significantly affected the compression-set [51]. Therefore, a hot-pressing time of 60 min was selected as the optimal condition.

**Figure 4:**
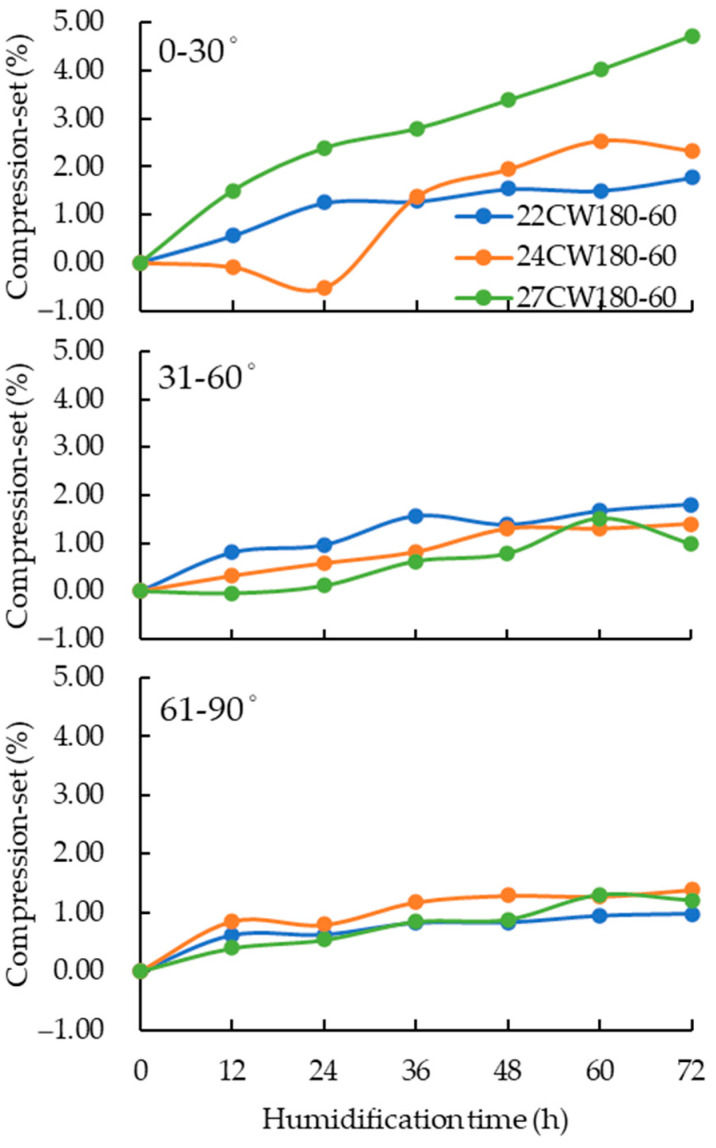
Compression-set of different annual ring angles.

Another factor that affects the compression-set of compressed wood is the annual ring angles. Zhang [16] classified annual ring angles into 0–30°, 31–60°, and 61–90° and found that the compression-set of annual ring angles of 61–90° was the lowest. The compression-set was compared with different annual ring angles under hot-pressing at 180 °C for 60 min. Figure 4 shows that the compression-set becomes more obvious as the annual ring angles increased.

The compression-set of annual ring angles of 0–30° measured by specimens with different thicknesses of 22, 24 and 27 mm was 1.78, 2.33 and 4.73% respectively, and compression-set of annual ring angles of 31–60° was 1.81, 1.39 and 0.98%, while the compression-set of annual ring angles of 60–90° was only 0.98, 1.39 and 1.21%. This could show that compression-set of annual ring angles of 0–30° was higher than other specimens and the highest compression rate of 33% of annual ring angles of 61–90° was 74% lower than that of annual ring angles of 0–30°. This phenomenon was related to the direction of transverse compression. During the compression process, the radial direction was destroyed by the shear stress generated by the sliding of the early and late wood, which made wood prone to cell cracking between the early and late wood during the loading process [52]. Therefore, to obtain the integrity of specimens after compression, the specimens with an annual angle ring of 61–90° was used for further experiment.

### 3.3. Drying-Set Treatment

#### 3.3.1. Compression-Set of Drying-Set Treatment

**Figure 5:**
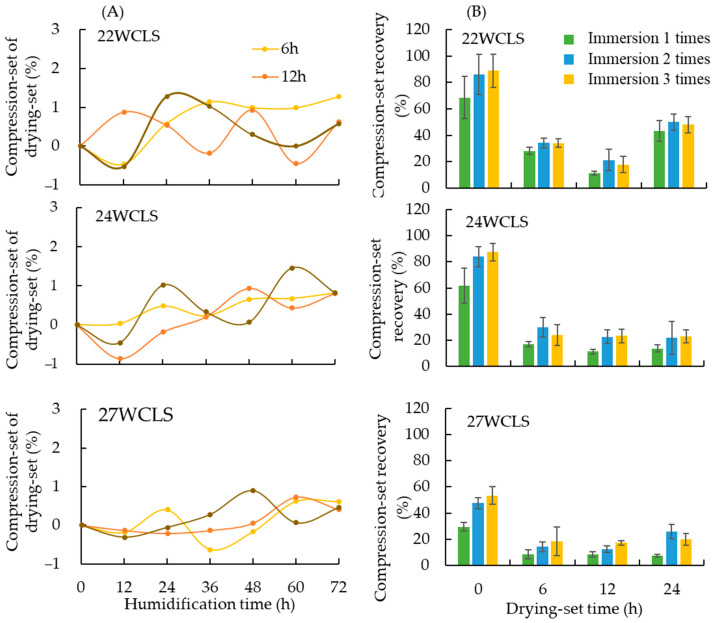
(**A**) Set-recovery of WCLS and (**B**) Compression-set recovery of WCLS.

After 72 h of testing in a constant temperature and humidity room, the results shown in Figure 5 indicated that the compression-set after the drying-set treatment was between 0.40% and 1.28%, which was higher than the specimens without drying-set treatment. The results revealed that after the drying-set treatment, the compression-set is reduced. For example, Esteves et al. [17] proposed that heat treatment after densification was the best treatment procedure.

Our results indicated that there was no significant difference in compression-set between the drying-set treatment times of 12 and 24 h, but the compression-set of 6 h was higher. Under different compression thickness conditions, the compression-set of drying-set treatment times of 6, 12, and 24 h ranged from 0.61 to 1.28%, 0.40 to 0.63%, and 0.45 to 0.82%, respectively. It can be observed that after prolonged heat treatment, the recovery rate decreases to below 1%, which is consistent with previous results [27].

The increasing awareness of environmental protection has made energy-saving an important topic of discussion. To achieve energy-saving benefits during the development of WCLS, a drying-set condition was chosen with a treatment time between 12 and 24 h, where compression-set was low and the difference was not significant. Selecting a shorter drying-set time during the development of WCLS can reduce energy consumption, and so a drying-set time of 12 h is recommended. In Figure 5A, 22WCLS, 24WCLS, and 27WCLS refer to specimen thickness-WCLS, and the legend indicates the drying-set time.

#### 3.3.2. Compression-Set Recovery

After hot pressing, the tested material was prone to rebound problems caused by residual stress. In order to reduce the occurrence of these problems, heat treatment was used to break the bonds of hemicellulose in the wood, with slight cracking of lignin, resulting in a decrease in cell hygroscopicity and improved dimensional stability [9,15,17]. The drying-set treatment involved drying at a stable temperature and continuous pressure for a long time to reduce the moisture content and release the residual stress after compression, effectively reducing the recovery [27].

The average compression-set recovery of WCLS after drying-set treatment under different development conditions was shown in Figure 5B The results showed that the average compression-set recovery of WCLS was higher than that of compressed wood specimens (22CW180-60, 24CW180-60, and 27CW180-60) with the same thickness, and the difference was significant. As shown in Figure 5A, the compression-set recovery tends to decrease with the increase of the compression-set rate. This result may be related to the state of the cell wall after compression. As the compressed thickness increases, the compressed area of the cell wall also increases, which may lead to crushing of the cell wall. Navi and Heger [10] reported that wood undergoes transverse densification, temperature, water, and compressive load to eliminate voids and produce compression forming.

The compression-set recovery after 6 h of drying-set treatment was slightly higher than that of 12 h, and the standard deviation was different for different compression-set rates, indicating that the effect was uneven only after 6 h of drying. However, the compression-set recovery after 24 h of drying-set treatment was not significantly different from that of 12 h, but its standard deviation was also relatively uneven. This suggests that the compression-set recovery after 12 h of drying-set treatment was low. The compression-set recovery for different compression thicknesses (22 mm, 24 mm, and 27 mm) after three immersion cycles was 17.84%, 23.34%, and 17.35%, respectively. By comparing with the compressed wood specimens (control group), the effect of the 12-h drying-set treatment was better than that of 6 and 24 h, with a compression-set recovery rate of 67–80%, while those of 6 and 24 h were between 61–72% and 45–73%, respectively. This result was consistent with the finding by Esteves et al. [17] that compression-set recovery dropped to 15–30% after three cycles of immersion for the test material subjected to heat treatment after densification. As the number of cycles of immersion increased and heat treatment time increased, the compression-set recovery of the test after densification before heat treatment tended to decrease. Inoue et al. [11] reported that to obtain a lower compression-set recovery, a higher temperature or longer treatment time was required. Therefore, based on the compression-setting recovery results, a drying-set treatment time of 12 h should be the best choice for the development conditions.

### 3.4. Basic Properties of WCLS

#### 3.4.1. Moisture Content, Density, Compression-Set and Weight Loss

The Japanese cedar specimen (18JC) in the control group and the WCLS with different thicknesses that underwent drying-set treatment after hot pressing were placed in a constant temperature and humidity room for over three weeks, and their moisture content was measured and recorded in Table S1. The moisture content of the Japanese cedar in the control group was 10.78%, while the moisture content of the WCLS with different compression-set rates ranged from 5.18–6.13%. The results indicated that the heat treatment after densification reduced the moisture content of the wood compared to the control group. When wood is heated at high temperatures, its chemical composition undergoes structural changes, which can increase the hydrophobicity of the cells and reduce the hygroscopicity of the wood [8].

As the compression-set rate increased, its density also increased. When the compression-set rate was 17.96%, its apparent density was 13% higher than that of the control group, and when the compression-set rate increased to 24.90%, it increased by 18%. When the compression-set rate was 33.22%, it increased by 26%. This result was consistent with the result of Zhang [16]. After compression, the densities with compression rates of 10 and 30% also increased by 7.3% and 42.3% compared with those without compression.

#### 3.4.2. Density Profile

The results of density profile were shown in Figure S5. The surface density of specimens with compression-set rates of 17.96%, 24.90%, and 33.22% was found to be 861.17 kg/m^3^, 715.34 kg/m^3^, and 567.84 kg/m^3^, respectively (Figure S5). The results also indicated that the higher the compression thickness, the higher the surface density. The development of WCLS achieved the effect of only surface densification, according to Shiu et al. [39]. They proposed that surface densification could effectively reduce the recovery rate, which was only 0.5–0.7% of the thickness. Kobayashi [53] reported that the technology of wood surface densification can solve the problems of low surface hardness and wear resistance of low-density wood.

### 3.5. Surface Properties of WCLS

#### Scanning Electron Microscope

**Figure 6:**
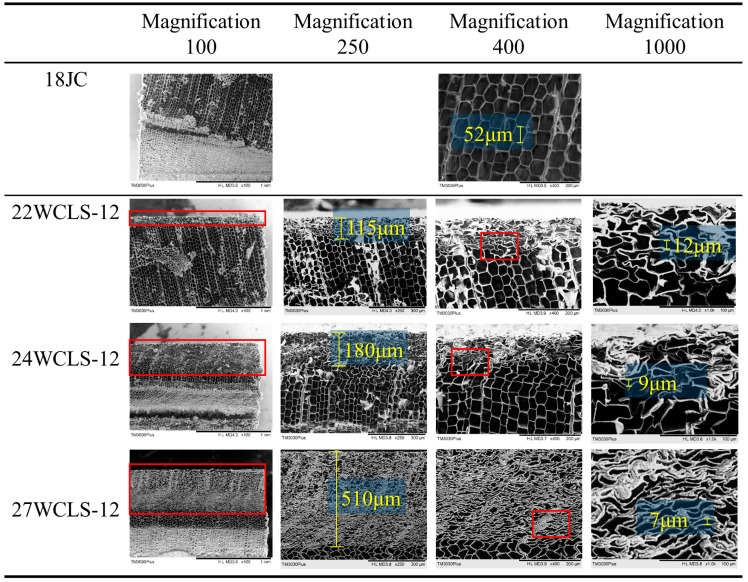
SEM photographs with different times for control group and WCLS.

Scanning electron microscopy was used to observe the cell walls of the control group and the WCLS (Figure 6). The results revealed that the densified part of the surface cells of the WCLS increased with the compression-set rate. The densified thicknesses of the development conditions of 22WCLS-12, 24WCLS-12, and 27WCLS-12 were 115 μm, 180 μm, and 510 μm, respectively. To further observe the cell walls, a magnification of 400 was used. The cell walls in the control group appeared hexagonal, while the WCLS at a compression-set rate of 33% showed a compacted and slightly curved shape that stacked to form a densified zone. The WCLS at a compression-set rate of 17.96% showed a compressed cell shape with a larger cell lumen space. Müller et al. [23] and Tu et al. [25] proposed that after high-temperature compression, the cell walls were softened by the influence of pressure and temperature, and the cell walls exhibited a rubbery state. This allows the cell walls to present a viscoelastic buckling state without rupture, and the volume of the void area is uniformly reduced.

To further understand the differences in cell size in the compression direction of WCLS, we observed the specimens at a magnification of 1000 and selected relatively obvious and complete cells in Figure 6 for measurement. The compression thickness ranged from low to high of the cell thicknesses were about 12, 9, and 7 μm, respectively, and the cell walls were not broken. We inferred that high temperature softens the cells, allowing them to bend without breaking and increasing the surface density. Zhang [16] has pointed out that mechanical properties such as hardness, modulus of rupture (MOR), and modulus of elasticity (MOE) increase with increasing wood density after compression. Therefore, densification of material with a higher density can be achieved.

### 3.6. Physico-Mechanical Properties of WCLS

#### 3.6.1. The Dimensional Stability

The results of dimensional stability of the compression wood of the control group and WCLS were shown in Table S2. After the drying-set treatment, the water absorption percent (WAP) decreased compared with the control group, and the longer the drying- set time, the WAP of WCLS was slightly decreased. Observing the development conditions of different compression-set rates, the WAP of specimen of 22WCLS-12 and 24WCLS-12 were significantly different from those of the control group with the same compression-set rate. Although there was no significant difference between specimen of 27WCLS-12 and the control group with the same compression-set rate, it still had a decreasing trend. This could be attributed to the degradation of hemicellulose after the wood was subjected to high temperature, which reduced the OH group that lead to reduction of water absorption of wood [26]. It was also known that as the compressed thickness increased, its water absorption tends to decrease.

In terms of the volumetric swelling coefficient (S), it can be observed that compared to the control group, the S of each WCLS that underwent drying-set treatment after compression had a decreasing trend. For the different development conditions of 22WCLS-12, 24WCLS-12, and 27WCLS-12, the reduction was 32%, 6%, and 18% lower than the control group. For the results of ASE, it can be seen that the WCLS specimens tended to increase. The results showed that drying-set treatment after hot pressing was beneficial to dimensional stability. However, the effectiveness of dimensional stability tended to decrease slightly with increasing compression-set rate. Sandberg and Johansson [27] proposed the drying-set method in which the moisture content drops rapidly during the drying process, and the stress stored in the cells will gradually disappear during compression. Therefore, the results showed that the developed specimen had good antiswelling efficiency. Shi et al. [54] addressed the issue that thicker wood specimens were more prone to fracture during compression. According to our results, we speculated that the antiswelling efficiency performance of the higher compression-set rate was poorer, possibly because the cell wall may be partially ruptured during the compression process or the internal storage stress after compression was not completely released.

On the other hand, the hygroscopicity results were shown in Table S3. After compression, its EMC was only 6.44–7.53%, but after drying-set treatment, it decreased to 5.04–6.58%, and compared with the same compression thickness, there was a significant difference between specimen w/wo drying-set treatment, which also indicated that after a long period of drying-set treatment, the hygroscopicity could be effectively reduced. Kuo and Lu [6] indicated that during the long-term heat treatment of wood, the wood was heated more uniformly, and the pyrolysis of hemicellulose or the reaction of lignin derivatives and cellulose produced ether bonds, while the hydrophilic OH group reduced, so that the hygroscopicity decreased.

The result of MEE could also show a slightly decrease in an environment with high relative humidity. However, increasing the drying-set treatment time increased MEE. For the same compression-set rates of 17.96% and 24.90%, it was observed that there was no significant difference between the drying-set time of 12 and 24 h, but both of them had significant differences compared with the one at 6 h. Kuo and Lu [6] proposed that the EMC of heat-treated Japanese cedar would decrease with increasing heat treatment time, while moisture excluding efficiency increased. The results of present study were consistent.

To sum up, drying-set treatment after hot pressing could reduce WAP, S and EMC, and then increase ASE and MEE. After the wood was heated, the result of the reduction of the hydrophilic group, the cross-linking reaction of cellulose and lignin, and long-term heat treatment to locally reduce the internal storage stress of the wood could change the hygroscopicity of the wood cells, thereby increasing the dimensional stability.

#### 3.6.2. Bending Strength

Tu et al. [25] suggested that the physical and mechanical properties of compressed wood increase with its density after compression, including hardness, MOR, and MOE. Similarly, Shiu et al. [39] found that hot-pressed wood could increase MOR by 30–130% and MOE by 25–70%. In the present study, only the surface layer of the test material was compressed during the hot-pressing process, forming a board structure with a higher density surface layer and lower density in the middle.

Table S4 showed that compared with the control group, the MOR value of Japanese cedar increased by 9.98, 13.39, and 20.87%, respectively, at compression-set rates of 17.96, 24.90, and 33.22% of WCLS. Similarly, MOE increased by 9.87, 17.37, and 22.40%, respectively. It is evident that the MOR and MOE of the WCLS improved with an increase in compression-set rate, which is consistent with O’Connor’s [19] findings. This trend is roughly proportional to the increase in wood density. Furthermore, there was an upward trend in MOR with an increase in compression-set rate, but there was no significant difference between the control group and each WCLS. Tu et al. [25] observed through microscopic observation that the volume of the intracellular void area was uniformly reduced, which plays a critical role in improving the mechanical properties of compressed wood.

#### 3.6.3. Surface Hardness

Kobayashi [53] suggested that wood surface densification technology can address issues related to the low surface hardness and wear resistance of low-density wood. In the present study, surface hardness increased with the thickness of compression after densification, as indicated in Table S5. In comparison with the control group of Japanese cedar, when the compression-set rate was 17.96, 24.90 and 33.22% of WCLS, the hardness values increased by 20.09, 30.85 and 40.22%, respectively. These results showed that the higher the compression-set rate, the greater the hardness. Blomberg et al. [20] pointed out that the main advantage of wood compression was the increase in wood mechanical properties due to the increase in density, which in turn led to an increase in hardness and bending strength. This result is also consistent with Zhang’s [16] findings that the Brinell hardness of test materials increased significantly as the compression rate increased.

### 3.7. Thermal Properties of WCLS

#### 3.7.1. The Thermogravimetric Loss

**Figure 7:**
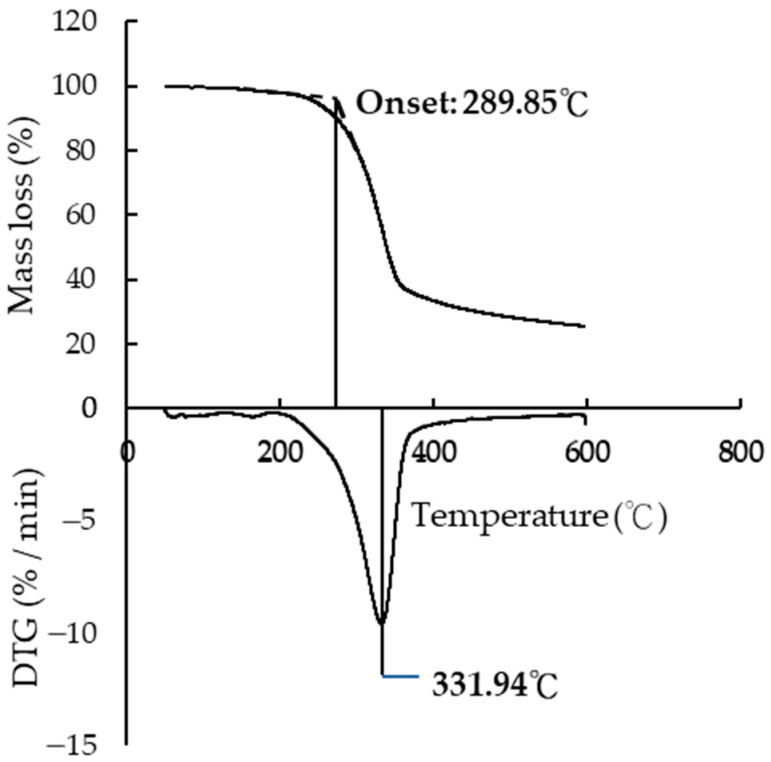
TGA and DTG curves of control group.

The main pyrolysis temperature and onset reaction temperature of the control group Japanese cedar were analyzed using thermogravimetric loss. The changes in the thermogravimetric loss were analyzed using TGA and DTG (Figure 7). The free water in the wood was lost first through dehydration when heated to 100 °C. Hemicellulose underwent thermal decomposition at temperatures between 200–260 °C, while cellulose decomposed at temperatures between 240–250 °C, and lignin underwent thermal degradation at 450 °C and above. Munir et al. [55] also noted that the weight loss in the first stage was due to water volatilization, while the second stage was the degradation caused by oxidation.

As shown in Figure 7, the onset temperature of the Japanese cedar reaction was 289.85 °C, with a weight loss rate of 15.17%. Furthermore, analysis of the DTG curve revealed that the maximum thermal decomposition temperature was 331.94 °C, with a loss rate of 43.28%, which was consistent with previous results [56]. The specimen started to undergo slight pyrolysis at 200 °C, which was associated with hemicellulose. Kamdem et al. [57] indicated that hemicellulose would undergo pyrolysis at 180 °C.

#### 3.7.2. Glass Transition Temperature

Hsu [58] stated that materials exhibit different states at different temperatures. In the case of wood, it shows thermal softening after being subjected to high temperatures. This process involves a state change of wood from elastic to plastic, and the temperature of the state change is known as the glass transition temperature (*Tg*). Hemicellulose has a *Tg* range of 167–217 °C, lignin has a range of 134–235 °C, and cellulose has a range of 231–253 °C [16,49].

**Figure 8:**
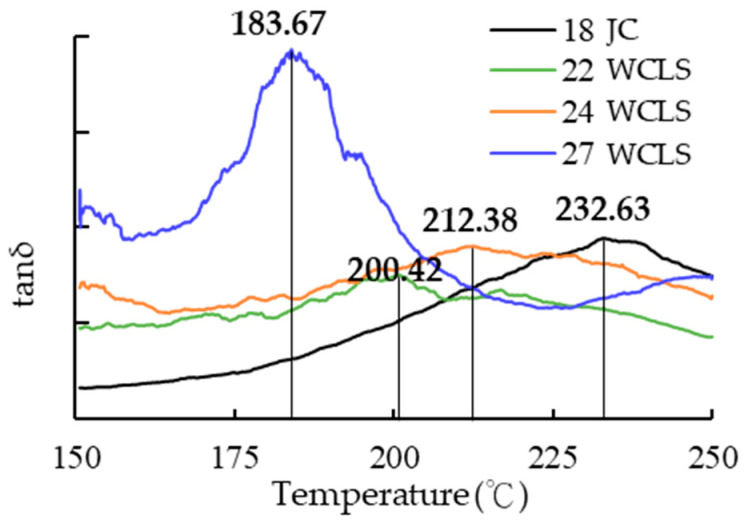
Loss tangent of control group and WCLS.

When the temperature ranges between 150–180 °C, the storage modulus (E′) and loss modulus (E″) of the wood are low. However, as the temperature increases beyond 180 °C, the E′ and E″ begin to increase, leading to an increase in Tan δ. At this point, the specimen begins to soften. When E′ decreases, E″ remains in a high state, causing a peak in Tan δ, which represents *Tg*. However, since wood is composed of various polymers and has different amounts of crystalline regions in cellulose, it affects *Tg*. Therefore, the *Tg* of specimens of 22WCLS-12 (200.42 °C), 24WCLS-12 (212.38 °C), and 27WCLS-12 (183.67 °C) was observed to determine if they were in a plastic state. Figure 8 showed that the *Tg* range values of Japanese cedar of the control group and different development conditions. The results of *Tg* analysis also showed that as the compression-set rate increased, it moved to the lower temperature region.

Kuo and Lu [6] pointed out that hemicellulose mainly consists of various types of saccharides, most of which are non-crystalline regions and contain more free hydroxyl groups. During heat treatment, the thermal degradation of hemicellulose results in a reduction of the hydroxyl group

It is evident that after high-temperature treatment of wood, the *Tg* moves to the low-temperature region due to the degradation of hemicellulose, which is mostly non-crystalline components, achieving the effect of easier thermal softening. Chang et al. [59] proposed that after Japanese cedar was subjected to heat treatment with high temperature at 150–230 °C, the wood composition would be changed.

### 3.8. Thermal Conductivity of WCLS with Different Compression-Set Rates in Atmospheric Environment

Suleiman et al. [60] noted that the thermal conductivity of wood was influenced by many factors, and the density of wood plays an important role. The amount of porosity, as well as the media existing in the pores and their distribution, affect the thermal conductivity of the wood. In this experiment, different compression-set rates of WCLS were analyzed, and it was found that as the compression-set rate increased, the thermal conductivity tended to decrease in the atmospheric environment (Table S6). The thermal conductivity of different compression-set rates of 17.96, 24.90, and 33.22% compared with the Japanese cedar of the control group decreased by 18.41, 2.50 and 23.60%, respectively. The thermal conductivity of WCLS may also be affected by the different thermal conductivity in the layered structure. The structure of WCLS is similar to that of engineered flooring, and the thermal conductivity of solid wood flooring is higher than that of modified engineered flooring. The thermal conductivity of engineered flooring is lower than that of solid wood flooring.

### 3.9. Effects of Different Temperature and Humidity on Thermal Conductivity and Electricity Energy Consumption of WCLS

**Figure 9:**
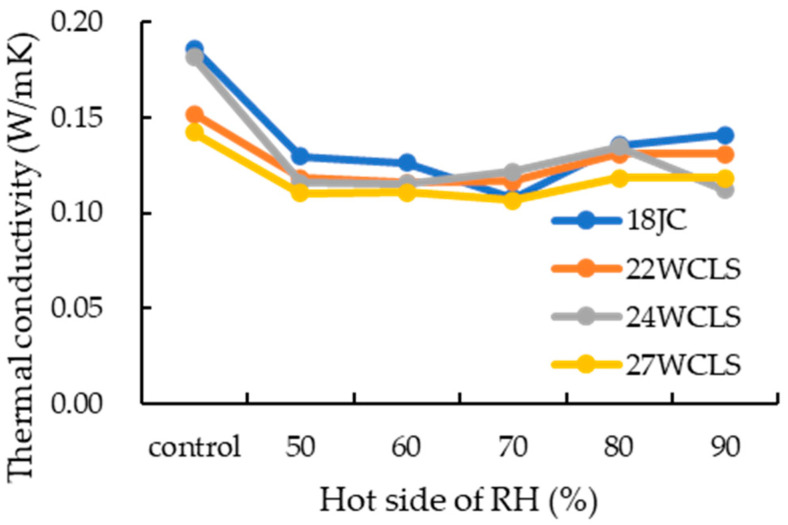
Thermal conductivity of control group and WCLS with different conditions.

Seo et al. [36] noted that thermal conductivity increases with density and decreases with porosity. Therefore, higher density wood tissue has higher thermal conductivity. Additionally, Yu [46] found that the thermal conductivity of Japanese cedar increased with relative humidity, with values of 0.096, 0.097, 0.101, 0.106, and 0.112 W/mK at relative humidities of 50, 60, 70, 80 and 90%, respectively. As shown in Figure 9, under the same temperature and humidity conditions on the hot side, there was no significant difference in the thermal conductivity of each WCLS compared to control group. However, as the compression-set rate increased, the thermal conductivity tended to decrease. At a compression-set rate of 33.22%, thermal conductivity was 14.8% lower than that of the control group. Thus, the compression-set rate had an influence on thermal conduction. As the relative humidity of the hot side increased, the thermal conductivity tended to increase, with a more significant decrease in thermal conductivity at relative humidities of 80–90%. Therefore, it could be inferred that due to the rise in relative humidity, the water in the pores of the control group increased, which increased the moisture content of the wood and resulted in faster thermal conduction. Liao [31] and Suleiman et al. [60] noted that air has low thermal conductivity (~0.023 W/mK), and since wood is a porous material, most of the pores are filled with air, leading to lower thermal conductivity. When the relative humidity of the hot side was 90%, there was a significant difference in thermal conductivity between WCLS and the control group, which suggests that the hydrophobicity improvement on the specimen after hot pressing and drying-set treatment reduced water absorption in high-humidity environments, resulting in lower thermal conductivity. There was no significant difference in the electricity energy consumption of WCLS with different compression thicknesses (data not shown), although Cuo [61] mentioned that electricity energy consumption of air conditioners could be saved by 18% compared to that without built-in insulation.

### 3.10. Effects of Simulated Outdoor Temperature and Humidity Changes on Thermal Conductivity and Electricity Energy Consumption of WCLS

**Figure 10:**
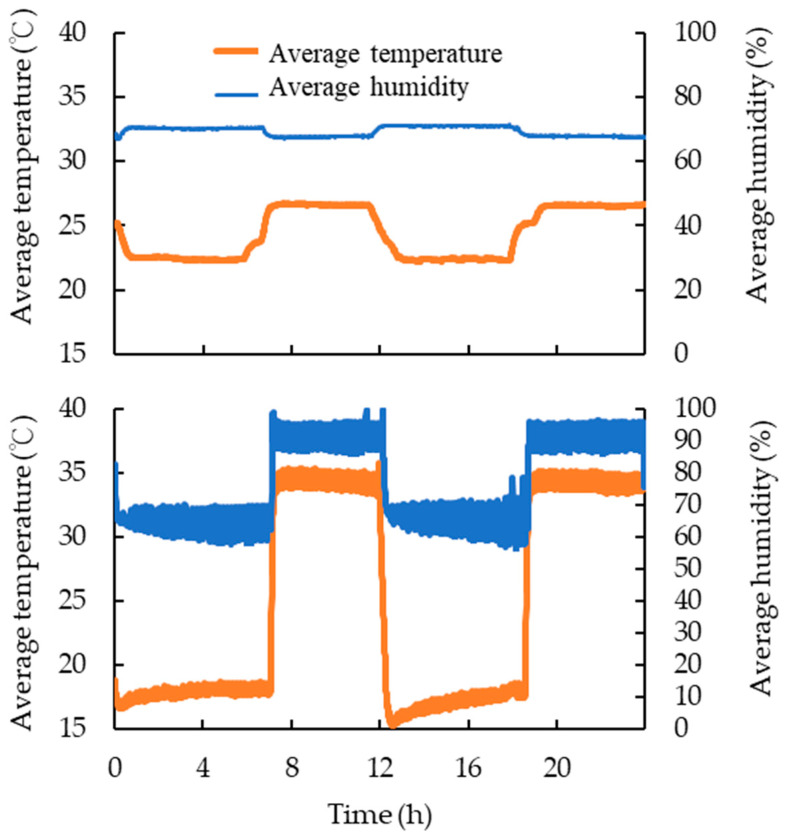
Cold (**UP**) and hot-side (**Down**) of temperature and relative humidity conditions.

Figure 10 shows the temperature and humidity conditions of the control group on the hot and cold side. The lower picture displays the average temperature and humidity changes on the hot side, while the upper picture shows the temperature and humidity changes on the cold side.

**Figure 11:**
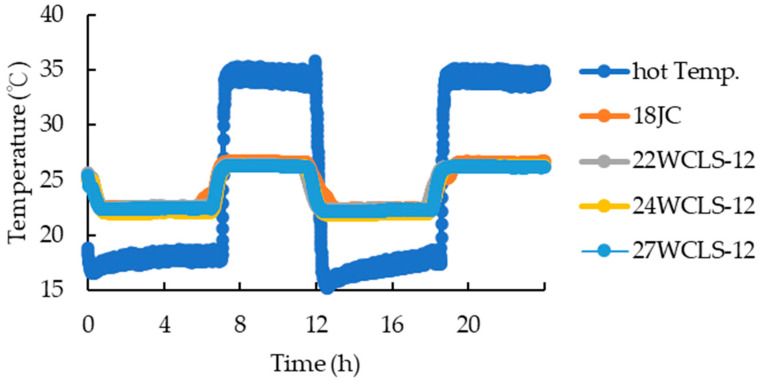
Cold and hot-side temperature of control group and WCLS.

Firstly, when observing the cold side temperature of the control group and WCLS (Figure 11), it was found that the cold side temperature of the control group was higher than that of WCLS. Under hot side conditions of 15 °C and relative humidity of 65%, the temperature of the cold side of the control group and each development condition of 22WCLS-12, 24WCLS-12 and 27WCLS-12 were 22.65, 22.57, 22.11 and 22.39 °C, respectively. Moreover, comparing the temperature on the cold side of the control group, each WCLS decreased by 0.34, 2.36 and 1.15%, respectively. This indicated that when the outdoor temperature was low, the temperature of the cold side of WCLS was closer to the set temperature of 25 °C than that of the control group, implying that WCLS had better thermal insulation. However, under hot side conditions of 35 °C and 90% relative humidity, the temperature on the cold side of the control group and each WCLS was 26.38, 26.07, 26.03 and 26.03 °C, respectively. Compared to the cold side temperature of the control group, the WCLS development conditions 22WCLS-12, 24WCLS-12 and 27WCLS-12 decreased by 1.16, 1.33 and 1.33%, respectively. The trend was the same under low temperature and humidity conditions on the hot side, and with an environment of higher temperature and humidity, the lower temperature of the cold side of WCLS was more clearly observed.

**Figure 12:**
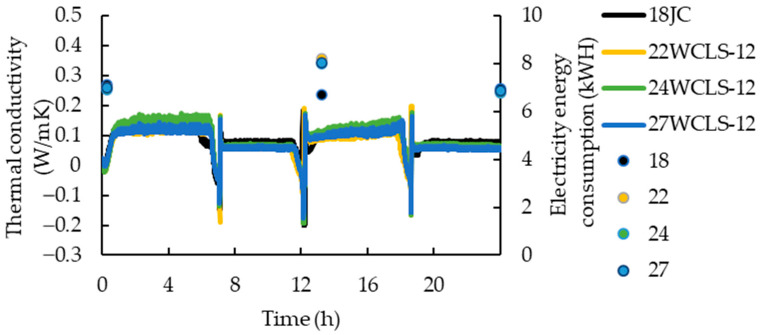
Thermal conductivity and cumulative electricity energy of control group and WCLS.

Under conditions of high temperature and humidity on the hot side, it could be observed that the thermal conductivity of the control group was higher than that of WCLS. The thermal conductivity values were 0.1037, 0.0802, 0.0926 and 0.0814 W/mK, respectively. Compared to the control group, the WCLS with low to high compression-set rates decreased by 22.52, 10.67 and 21.47%, respectively (Figure 12). Yu [46] and Liao [31] noted that a lower thermal conductivity resulted in a higher thermal resistance value, which indicated better thermal insulation of the material. The thermal conductivity measured on the cold side was lower for WCLS than that of the control group when used to block the cold and hot sides, and the temperature of the cold side also exhibited a downward trend. Thus, it could be inferred that the WCLS developed by the research institute helped to reduce the thermal conductivity to achieve the effect of maintaining indoor temperature.

Regarding electricity energy consumption, the average consumption of the control group and the different development conditions 22WCLS-12, 24WCLS-12 and 27WCLS-12 were 30.50, 29.83, 29.57 and 29.4 kwH, respectively. Although the electricity energy consumption of the control group was relatively high, it tended to decrease as the compression thickness increased, which was consistent with the results of thermal conductivity. When observed under different hot side temperature and humidity conditions, the electricity energy consumption of the control group was almost always higher than that of WCLS. Liao [31] pointed out that wood as an interior decoration material could achieve effective thermal insulation and preservation, making the indoor environment less susceptible to the external environment. With indoor air-conditioning, the set temperature could be easily maintained, and it was not necessary to continue operating to reach the set temperature, achieving a power-saving effect. The aforementioned results were related to the water absorption of the material. The decrease in surface roughness and increase in contact angle after hot pressing and drying-set treatment resulted in better surface hydrophobicity and moisture exclusion, making the material able to withstand high temperatures and humidity. In such environments, the material absorbed less moisture, leading to less water in the pores and, subsequently, a decrease in thermal conductivity. Therefore, in the cyclic test, as the compression-set rate increased, the thermal conductivity decreased, making it easier to maintain the set temperature and humidity on the cold side, thus reducing electricity consumption. The WCLS developed in this research can be obtained as a material with energy-saving benefits.

## 4. Conclusions

In summary, after the development of WCLS and its evaluation for dimensional stability, the density, surface properties, and physico-mechanical properties showed a relative increase trend. Regarding thermal conductivity, the surface hydrophobicity and moisture excluding efficiency were improved. This led to a reduction in thermal conductivity and subsequently in electricity energy consumption, resulting in energy-saving benefits. Therefore, the WCLS developed in this study is a material with high strength, stable dimensions, and energy-saving properties. Our findings provided additional insights that can benefit future forestry-related industries, and may promote the utilization of domestic wood resources in decorating industry.

## Figures and Tables

**Table 1:** Conditions of specimen preparation.

Thickness (mm)	Compress Mode	Temperature (°C)	Time (min)	Specimen Name ^c^
18 ^a^	-	-	-	18JC
22	CW ^b^	160	30	22CW160-30
			60	22CW160-60
		180	30	22CW180-30
			60	22CW180-60
		200	30	22CW200-30
			60	22CW200-60
24	CW	160	30	24CW160-30
			60	24CW160-60
		180	30	24CW180-30
			60	24CW180-60
		200	30	24CW200-30
			60	24CW200-60
27	CW	160	30	27CW160-30
			60	27CW160-60
		180	30	27CW180-30
			60	27CW180-60
		200	30	27CW200-30
			60	27CW200-60

^a^ Control group: Japanese cedar specimens; ^b^ Compress wood; ^c^ Specimen thickness: 22, 24, and 27 mm. Temperature: 160, 180, and 200 °C. Hot-pressing time: 30 and 60 min.

**Table 2:** Treatments of WCLS.

Size (mm)	Compression Mode	Temperature (°C)	Time (min)	Drying-Set Mode	Time (h)	Samle Name ^c^
22	WC ^a^	180	60	LS ^b^	0	22WCLS-0
					6	22WCLS-6
					12	22WCLS-12
					24	22WCLS-24
24	WC	180	60	LS	0	24WCLS-0
					6	24WCLS-6
					12	24WCLS-12
					24	24WCLS-24
27	WC	180	60	LS	0	27WCLS-0
					6	27WCLS-6
					12	27WCLS-12
					24	27WCLS-24

^a^ WC: hot-pressing mode; ^b^ LS: drying-set mode; ^c^ 22, 24, and 27: initial thickness before compression; CW: compression code; 160, 180, and 200: hot pressing temperature; 30 and 60: hot pressing time.

**Table 3:** Moisture content and density of different thickness specimen.

Specimens Thickness (mm)	Moisture Content (%)	Density (kg/m^3^)
18	10.78 (0.56) ^a(1)^	386.33 (0.04) ^ca^
22	9.77 (0.20) ^a^	427.39 (0.08) ^ab^
24	9.80 (0.98) ^a^	449.43 (0.04) ^bc^
27	10.05 (0.20) ^a^	506.98 (0.05) ^c^

*^(^*^1)^ Average (SD). Columns with different letters indicate significant differences (*p* < 0.05).

## Data Availability

The data presented in this study are available on request from the corresponding author.

## Supplementary Materials

The following supporting information can be downloaded at: https://www.mdpi.com/article/10.3390/polym15153208/s1, Figure S1: Japanese cedar boards were divided into three groups using different annual ring angle, Figure S2: Specimen processing; Figure S3: Sampling method of test specimens; Figure S4: Method of fixed specimen; Figure S5: Density profile of WCLS. Table S1: Moisture content, density, compression-set and weight loss of specimens; Table S2: Water absorption percent, volumetric swelling coefficient and antiswelling efficiency with different compression-set WCLS; Table S3: Equivalent moisture content and moisture excluding efficiency with different compression thickness wood compression layered structural materials; Table S4: Modulus of rupture and Modulus of elasticity of different compression-set rate WCLS; Table S5: Surface hardness of different compression-set WCLS; Table S6: Thermal conductivity of WCLS.
