# Supplementary material for: Evaluation of Physico-Mechanical Properties and Thermal Conduction to Energy-Saving Effects of Wood Compression Layered Structural Materials
Source: Polymers (Basel). 2023 Jul 28;15(15):3208. doi: 10.3390/polym15153208 (PMC10421106; doi:10.3390/polym15153208)
Supplement: Supplementary file 1 [file polymers-15-03208-s001.zip › polymers-2467496-supplementary.pdf]

Evaluation of Physico-mechanical Properties and Thermal Conduction to Energy-saving Effects of Wood Compression Layered Structural materials

Yu-Hui Chen[1#], Shang-Tse Ho[1#], and Han-Chien Lin [1,*]

[1] Department of Wood Based Materials and Design, National Chiayi University, Chiayi 600, Taiwan.

* Correspondence: alexhlin@mail.ncyu.edu.tw; Tel.: +886-5-271-7100; Han-Chien Lin

[#] These authors contributed equally to the work.

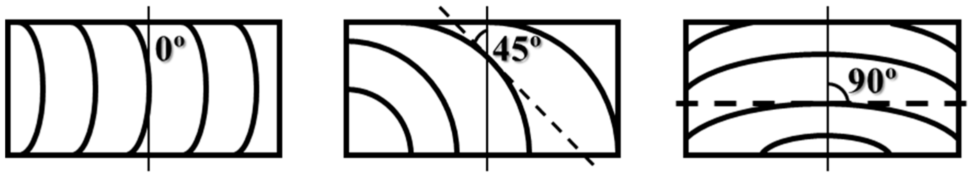

Figure S1. Japanese cedar boards were divided into three groups using different annual ring angle.

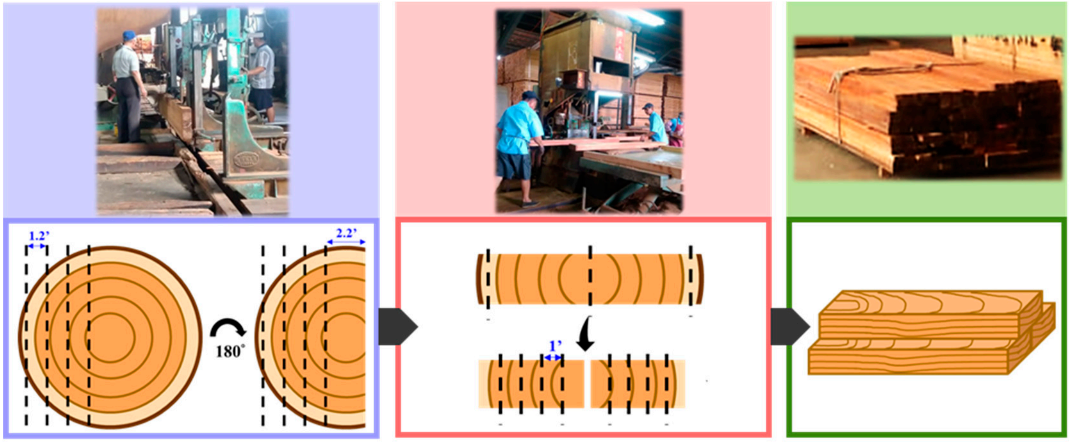

Figure S2. Specimen processing.

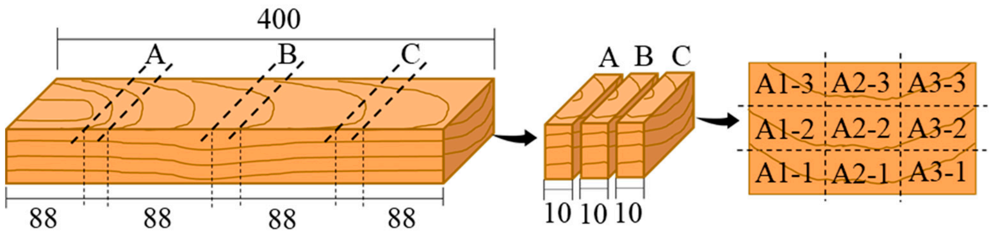

Figure S3. Sampling method of test specimens.

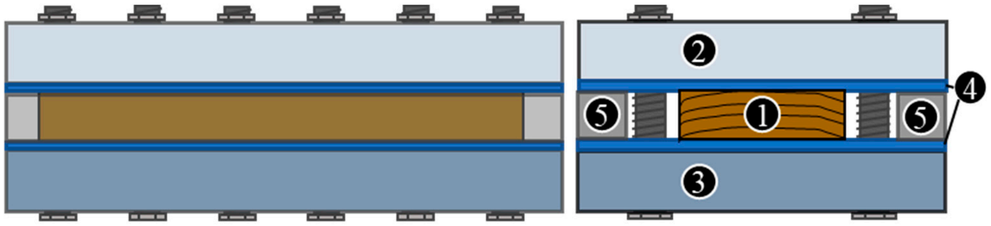

❶ WCLS specimens；❷ Iron plates with threaded holes
❸ Iron plates with non-threaded holes；❹ Stainless plate
❺ Stop bar with thickness 18 mm

Figure S4. Method of fixed specimen.

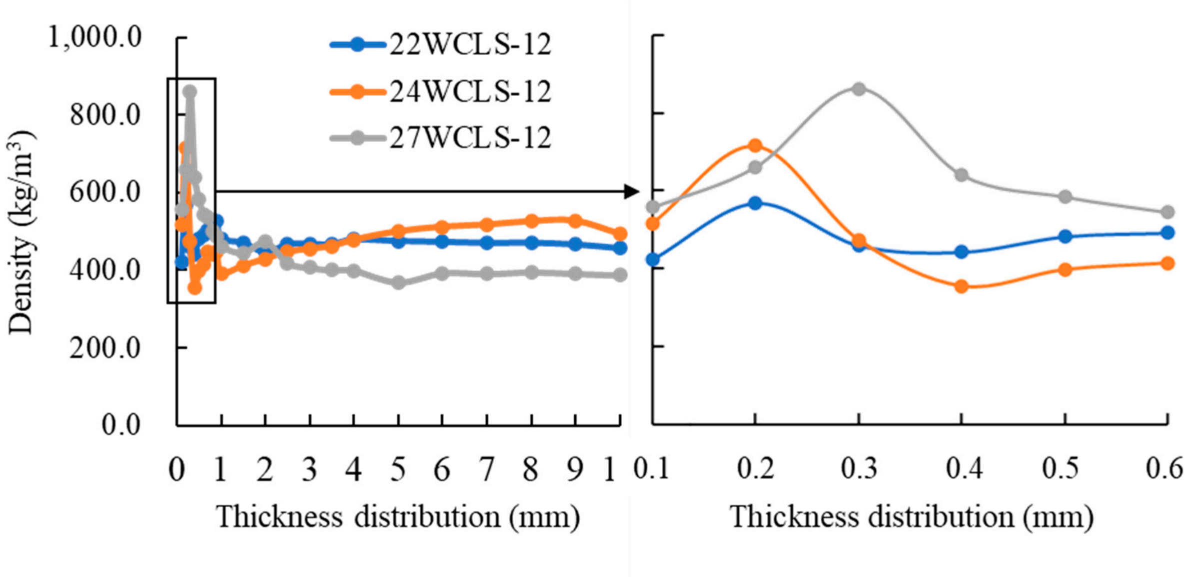

Figure S5. Density profile of WCLS.

Table S1 Moisture content, density, compression-set and weight loss of specimens with different manufacturing conditions

Development conditions	Moisture content (%)	Compression-set rate (%)	Density (kg/m^3)	Weight loss (%)
18JC	10.78 (0.56)$^{a1)}$	-	386.33 (0.04)^{ca}	-
22WCLS-12	6.13 (0.66)^{b}	17.96 (0.41)	427.39 (0.08)^{ab}	0.88 (0.59)^{a}
24WCLS-12	5.81 (0.34)^{b}	24.90 (0.18)	449.43 (0.04)^{bc}	1.02 (0.61)^{a}
27WCLS-12	5.08 (0.60)^{b}	33.22 (0.25)	506.98 (0.05)^{c}	1.23 (0.84)^{a}

[1)] Average (SD). Letters are the test results of Duncan's single factor variance at the significance level of 5%. The same lowercase letters mean that there is no significant difference in between the development conditions and the measurement items.

Table S2 Water absorption percent, volumetric swelling coefficient and antiswelling efficiency with different compression-set WCLS

Development conditions	Cr (%)[1)]	WAP(%)	S(%)	ASE(%)
22CW180-60	17.96	202.78 (9.43)^{a}[2)]	27.17 (3.63)^{ha}	-
22WCLS-6		198.33 (3.62)^{ab}	17.22 (3.62)^{b}	34.38 (13.33)^{a}
22WCLS-12		188.77 (7.56)^{bc}	18.55 (2.49)^{b}	36.46 (6.83)^{a}
22WCLS-24		178.77 (3.62)^{c}	16.48 (2.56)^{b}	39.32 (9.43)^{a}
24CW180-60	24.90	185.46 (2.89)^{a}	28.63 (1.88)^{a}	-
24WCLS-6		170.48 (1.05)^{b}	23.72 (1.00)^{b}	15.67 (3.30)^{a}
24WCLS-12		178.37 (5.97)^{c}	26.78 (3.33)^{ac}	16.07 (6.77)^{a}
24WCLS-24		178.90 (3.13)^{c}	24.68 (1.18)^{bc}	18.78 (4.12)^{a}
27CW180-60	33.22	177.22 (6.35)^{a}	29.04 (7.47)^{a}	-
27WCLS-6		176.03 (9.51)^{a}	27.33 (2.14)^{a}	8.20 (5.18)^{a}
27WCLS-12		169.28 (5.30)^{a}	23.72 (1.00)^{a}	13.20 (15.11)^{a}
27WCLS-24		173.73 (5.00)^{a}	27.88 (7.04)^{a}	12.59 (27.08)^{a}

[1)] Compression-set rate (Cr)

[2)] Average (SD). Columns with different letters indicate significant differences ($p < 0.05$).

Table S3 Equivalent moisture content and moisture excluding efficiency with different compression thickness wood compression layered structural materials

Development conditions	Cr (%)[1)]	40℃, 65%RH		40℃, 95%RH	
		EMC(%)	MEE(%)	EMC(%)	MEE(%)
22CW180-60	17.96	6.51 (0.16)[a2)]	-	16.42 (1.06)[a]	-
22WCLS-6		6.46 (0.70)[a]	7.94 (5.79)[a]	16.54 (0.37)[a]	2.65 (2.36)[a]
22WCLS-12		5.71 (0.51)[b]	17.81 (7.58)[ab]	13.93 (1.55)[b]	19.25 (3.32)[b]
22WCLS-24		5.04 (0.68)[b]	29.64 (5.69)[b]	13.87 (0.88)[b]	18.77 (7.31)[b]
22CW180-60	24.90	7.53 (0.67)[a]	-	18.69 (5.37)[a]	-
22WCLS-6		6.58 (0.28)[b]	14.44 (4.86)[a]	16.76 (0.75)[a]	11.67 (5.04)[a]
24WCLS-12		6.46 (0.51)[b]	17.16 (9.63)[a]	15.69 (1.09)[a]	19.58 (8.31)[b]
24WCLS-24		6.14 (1.00)[b]	25.47 (4.42)[a]	16.51 (0.16)[a]	13.18 (1.11)[ab]
22CW180-60	33.22	6.44 (0.46)[a]	-	15.01 (0.25)[a]	-
27WCLS-6		5.12 (0.46)[b]	16.76 (3.54)[a]	13.67 (0.88)[b]	10.16 (7.55)[a]
27WCLS-12		6.17 (1.07)[a]	17.27 (3.77)[a]	12.66 (4.26)[b]	15.18 (1.11)[a]
27WCLS-24		5.82 (0.26)[ab]	13.50 (2.37)[a]	15.30 (1.49)[b]	12.76 (1.87)[a]

1) Compression-set rate (Cr)

2) Average (SD). Columns with different letters indicate significant differences ($p < 0.05$).

Table S4 Modulus of rupture and Modulus of elasticity of different compression-set rate WCLS

Development conditions[2)]	Cr (%)[1)]	MOR (MPa)	Increase rate (%)	MOE (GPa)	Increase rate (%)
18JC	0	51.84 (4.40)[a2)]	-	4.99 (0.39)[a]	-
22WCLS-12	17.96	52.14 (7.22)[a]	9.98 (9.71)	5.50 (1.03)[a]	9.87 (11.92)
24WCLS-12	24.90	56.22 (8.76)[a]	13.39 (20.61)	5.86 (0.90)[a]	17.37 (18.03)
27WCLS-12	33.22	61.06 (6.66)[a]	20.87 (13.09)	6.11 (0.90)[a]	22.40 (17.97)

[1)] Compression-set rate (Cr).

[2)] Average (SD). Columns with different letters indicate significant differences ($p < 0.05$).

Table S5 Surface hardness of different compression -set WCLS

Development confitions	Cr (%)[1)]	Density (kg/m^3)	Hardness (N/mm^2)	Increase rate (%)
18JC	0	386.33 (0.04)^a	5.60 (0.65)^{a}[2)]	-
22WCLS-12	17.96	427.39 (0.08)^{ab}	6.73 (0.44)^a	20.09 (7.84)
24WCLS-12	24.90	449.43 (0.04)^{bc}	8.54 (0.54)^b	30.85 (9.65)
27WCLS-12	33.22	506.98 (0.05)^c	9.57 (1.48)^b	40.22 (9.92)

[1)] Compression-set rate (Cr)

[2)] Average (SD). Columns with different letters indicate significant differences ($p < 0.05$).

Table S6 Thermal conductivity of WCLS

development condition	Cr (%)[1)]	Moisture content (%)	Density (kg/m^3)	Thermal conductivity (W/mK)
18JC	0	10.78 (0.56)[a2)]	386.33 (0.04)[a]	0.1863 (0.0071)[a]
22WCLS-12	17.96	6.13 (0.66)[b]	427.39 (0.08)[ab]	0.1520 (0.0147)[b]
24WCLS-12	24.90	5.81 (0.34)[b]	449.43 (0.04)[bc]	0.1817 (0.0106)[a]
27WCLS-12	33.22	5.08 (0.60)[b]	506.98 (0.05)[c]	0.1423 (0.0137)[b]

[1)] Compression-set rate (Cr)

[2)] Average (SD). Columns with different letters indicate significant differences ($p < 0.05$).
